# A Rolling Circle Replication Mechanism Produces Multimeric Lariats of Mitochondrial DNA in *Caenorhabditis elegans*

**DOI:** 10.1371/journal.pgen.1004985

**Published:** 2015-02-18

**Authors:** Samantha C. Lewis, Priit Joers, Smaranda Willcox, Jack D. Griffith, Howard T. Jacobs, Bradley C. Hyman

**Affiliations:** 1 Department of Biology and Interdepartmental Graduate Program in Genetics, Genomics and Bioinformatics, University of California Riverside, Riverside, California, United States of America; 2 BioMediTech and Tampere University Hospital, University of Tampere, Tampere, Finland; 3 Estonian Biocentre, Tartu, Estonia; 4 Lineberger Comprehensive Cancer Center, University of North Carolina at Chapel Hill, Chapel Hill, North Carolina, United States of America; 5 Molecular Neurology Research Program, University of Helsinki, Helsinki, Finland; University of Cologne, GERMANY

## Abstract

Mitochondrial DNA (mtDNA) encodes respiratory complex subunits essential to almost all eukaryotes; hence respiratory competence requires faithful duplication of this molecule. However, the mechanism(s) of its synthesis remain hotly debated. Here we have developed *Caenorhabditis elegans* as a convenient animal model for the study of metazoan mtDNA synthesis. We demonstrate that *C. elegans* mtDNA replicates exclusively by a phage-like mechanism, in which multimeric molecules are synthesized from a circular template. In contrast to previous mammalian studies, we found that mtDNA synthesis in the *C. elegans* gonad produces branched-circular lariat structures with multimeric DNA tails; we were able to detect multimers up to four mtDNA genome unit lengths. Further, we did not detect elongation from a displacement-loop or analogue of 7S DNA, suggesting a clear difference from human mtDNA in regard to the site(s) of replication initiation. We also identified cruciform mtDNA species that are sensitive to cleavage by the resolvase RusA; we suggest these four-way junctions may have a role in concatemer-to-monomer resolution. Overall these results indicate that mtDNA synthesis in *C. elegans* does not conform to any previously documented metazoan mtDNA replication mechanism, but instead are strongly suggestive of rolling circle replication, as employed by bacteriophages. As several components of the metazoan mitochondrial DNA replisome are likely phage-derived, these findings raise the possibility that the rolling circle mtDNA replication mechanism may be ancestral among metazoans.

## Author Summary

Defects in the mitochondrial DNA (mtDNA) that encodes protein subunits of the respiratory complexes may cause severe metabolic disease in humans. Such defects are often caused by errors during mtDNA synthesis, motivating ongoing studies of this process. The nematode *Caenorhabditis elegans* has been proposed as a model for the study of mtDNA replication defects. Here we analyze the mechanism of mtDNA synthesis in the *C*. *elegans* gonad and demonstrate that it is unique among animals. Nascent worm mtDNA forms branched-circular lariat structures with concatemeric tails that we suggest would ultimately resolve into monomeric circles, the predominant molecular form identified by both transmission electron microscopy and two-dimensional gel electrophoresis. Our discovery that mtDNA replication in *C*. *elegans* does not faithfully model that in mammals is significant, because it demonstrates the breadth and evolutionary plasticity of the mechanisms that maintain this critical DNA among animals. Interestingly, the mtDNA replication mechanism within *C*. *elegans* is highly similar to that of bacteriophages, from which components of the mitochondrial DNA replisome are thought to be derived. Thus *C*. *elegans* may serve as a model for mtDNA synthesis as it occurred within ancient eukaryotes.

## Introduction

*Caenorhabditis elegans* is a ubiquitous model animal often employed in studies of aging and metabolic disease, processes intimately associated with mitochondrial health. However, comparatively little is known of mtDNA maintenance in this organism [1,2].

Early studies of mitochondrial DNA (mtDNA) replication in mammalian cultured cells supported a unidirectional strand displacement or ‘asymmetric’ model, producing partially single-stranded-DNA (ssDNA) intermediates [3,4]. More recently, strand-coupled ‘theta’ replication has been proposed [5], and support has also amassed for a temporally asynchronous mode of replication involving provisional RNA Incorporation ThroughOut the Lagging Strand (RITOLS), a model in which expanding replication bubbles contain RNA:DNA hybrid tracts [6]. The previously described animal mtDNA replication models share two features. First, initiation of replication relies on elongation from a transcript-primed displacement loop (D-loop). Second, each successful synthesis cycle from the circular template results in only two circular daughter molecules. The previous work on mtDNA synthesis has focused primarily on mammalian species; mtDNA maintenance elsewhere in the animal lineage remains poorly understood.

MtDNA is required for nematode development beyond the early larval stages, and perturbations causing mtDNA depletion during embryonic development commonly result in a larval arrest phenotype [7]. The mitochondrial complement in somatic cells of the adult nematode appears largely to result from distribution of the approximately 100,000 maternal mtDNA molecules throughout the embryo during development, precluding a need for the mitochondrial polymerase POLG-1 during development [8]. Moreover, mtDNA copynumber tends to fall in ageing worms, suggesting minimal turnover in the somatic tissues [8]. These findings are consistent with mtDNA replication occurring primarily in the adult gonad, with the integrity and quantity of mtDNA produced reflected in the subsequent generation [8]. The confinement of ongoing mtDNA replication to the germline makes *C*. *elegans* a convenient model for studies of mitochondrial genome synthesis and mtDNA replication defects [1,9].

**Fig 1:**
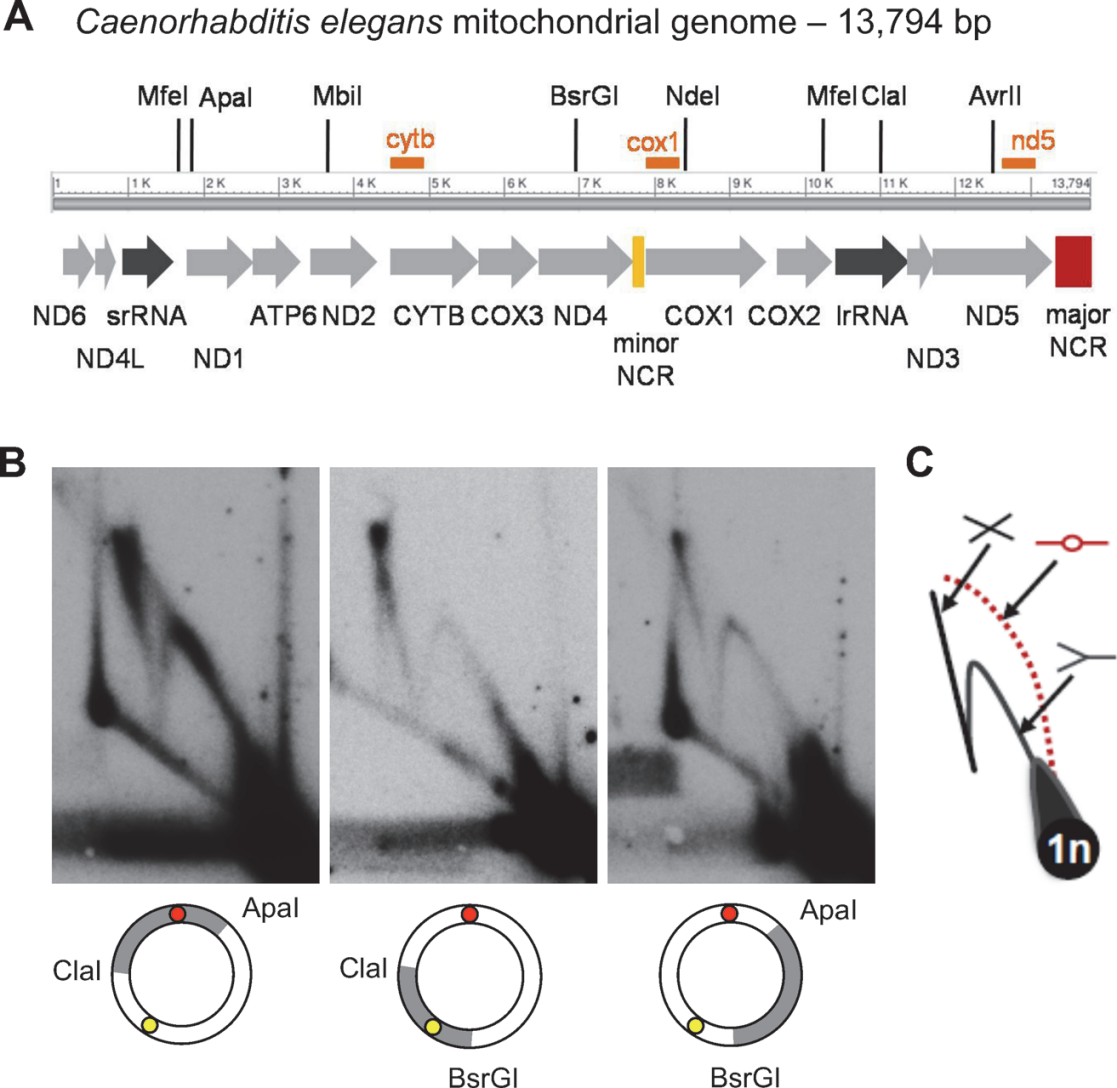
2DNAGE analysis of *C*. *elegans* mitochondrial DNA reveals prominent replication and recombination intermediates but no initiation bubbles. (A) Physical and transcriptional maps of the *Caenorhabditis elegans* mitochondrial genome, arbitrarily linearized for clarity. Arrows depict transcriptional orientation and relative lengths of protein coding genes (light grey arrows), large and small ribosomal RNA genes (dark grey arrows), and two NCRs (yellow and red bars). Hybridization probe locations are indicated by orange bars; see also Supplementary Information. (B) MtDNA isolated from a sucrose gradient-enriched mitochondrial preparation was restriction enzyme cleaved as indicated to generate 3.8 or 5 kb fragments that collectively cover the entire genome, subjected to 2DNAGE and probed to identify intermediates containing the 465 bp ‘major’ NCR (ClaI/ApaI digest; red circle); the 104 bp ‘minor’ NCR (BsrGI/ClaI digest; yellow circle); or a coding region only (ApaI/BsrGI digest). (C) A diagram of replication intermediate migration within 2DNAGE gels is depicted (counter-clockwise from lower right: 1n spot of linear monomer mtDNA fragments; Y-shaped replication forks; bubble-shaped initiation intermediates, in this case absent from the gels, hence depicted in red, with dotted line; cruciform recombination intermediates). Panels are representative of three independent experiments per fragment.

*C*. *elegans* mtDNA harbors two non-coding regions (NCRs), delimiting coding regions of 5.5 and 7.7 kb respectively (Fig. 1A; [10]). By analogy with the mammalian mtDNA organization, both NCRs have been proposed to play a role in *C*. *elegans* mtDNA replication, one as the first-strand origin (akin to the mammalian D-loop) and the other serving as a second-strand origin [1,7]. To test this assumption, we investigated the mechanism of *C*. *elegans* mtDNA replication *in vivo* and the possible function of the two NCRs therein.

## Results

### Non-coding mtDNA regions lack bubble origin activity

To determine if either NCR could function as a replication origin, we examined mtDNA fragments containing each one of the two NCRs for origin activity using two-dimensional neutral agarose gel electrophoresis (2DNAGE) [11]. Y arcs formed by progressing forks, as well as cruciform structures, were readily apparent (Fig. 1B, ClaI/ApaI and BsrGI/ClaI). Analysis of replication intermediates (RIs) derived from restriction fragments lacking both NCRs also revealed full Y arcs and cruciforms, consistent with active replication of the entire mtDNA (Fig. 1B, ApaI/BsrGI). However, a bubble arc indicative of theta-type replication initiation was not detected from any region of the genome, even after long autoradiographic exposures (S1A–S1E Fig.).

We next considered that first-strand replication initiation might occur from more than one site in the genome. Such low frequency bubble intermediates may go undetected by fragment 2DNAGE, as only a subset of intermediates are analyzed in each experiment [4,6,11]. To address this possibility, we performed 2DNAGE analysis after digestion with restriction enzymes cutting only once in the mitochondrial genome. We reasoned that analysis of RIs spanning the complete mtDNA would pool molecules containing D-loop initiation structures along a single arc regardless of initiation site, facilitating detection. Consonant with our 2DNAGE data on sub-genomic fragments, linearization and subsequent 2DNAGE of the full-length genome demonstrated clear Y and X shaped intermediates (S1F–S1J Fig.), yet no bubble arc was observed. We therefore conclude that initiation of *C*. *elegans* mtDNA synthesis does not involve the formation of a bubble intermediate at levels detectable by blot-hybridization.

To determine if molecular signatures of replication initiation, such as skewed nucleotide composition, are present in the non-coding region of *C*. *elegans* mtDNA, we conducted a bioinformatic analysis of cumulative GC skew (S1K Fig.; [12]). Unlike the D-loop regions of human and mouse mtDNA, our analysis demonstrates that the non-coding region suggested to harbor a D-loop in *C*. *elegans* is absent of local minima or maxima, considered features of origin and termination activity respectively. This finding is consistent with the lack of classic initiation (bubble) arcs on 2D-NAGE gels of replication intermediates.

Prominent initiation intermediates have been described in analyses of mtDNA isolated from dissected human, mouse and chick tissues [5,13,14]. However, in the worm the vast majority of mtDNA replication is expected to occur specifically in the germline. Therefore, we tested whether a minor fraction of bubble initiation structures from somatic cells would become detectable when germline development was blocked. We isolated mtDNA from synchronized *glp-4* mutants which exhibit deficient germline nuclei production at 25°C (allele *glp-4(bn2)*; [15]), and compared nematode cohorts reared at permissive and non-permissive temperature by 2D-NAGE. As expected, based on the work of Bratic et al [8] replication intermediates in *glp-4(bn2)* animals cultured at 16°C were comparable in structure and intensity to those detected in wildtype N2 animals. In contrast, at non-permissive temperature, i.e., in “gonadless” worms, RI levels relative to total mtDNA were dramatically decreased to near the limit of detection by Southern hybridization (S2A–S2B Fig.). Bubble intermediates were not detected on exposures ranging from 1 hour to 14 days, confirming the absence of replication elongation from a D-loop from both germline and post-mitotic cells. The lack of bubble intermediates effectively excludes strand-coupled initiation from a D-loop, i.e. the theta replication mode [4].

### Strand-synchronous replication of nematode mtDNA produces dsDNA replication intermediates

We next investigated whether *C*. *elegans* mtDNA RI structure was consistent with the asymmetric strand-displacement or RITOLS models of mtDNA replication. Temporally asynchronous replication of the two template strands is predicted to generate partially single-stranded RIs [4,16] and references therein]. In 2DNAGE, such ssDNA regions block endonuclease cleavage, producing slow-migrating Y arcs greater than twice the unit length fragment size, and render RIs sensitive to the action of the single-strand specific nuclease S1 [4]. In contrast, RNA:DNA hybrid-containing RIs typical of the RITOLS mode can be detected based on their sensitivity to degradation by RNase H, which exposes ssDNA regions rendered sensitive to S1 nuclease [17,18]. These treatments are expected to dramatically alter the electrophoretic migration properties of RITOLS intermediates in 2D gels [4,14]; such RNA:DNA hybrid-containing intermediates represent a transient step in replication, preceding synthesis of the definitive lagging strand.

**Fig 2:**
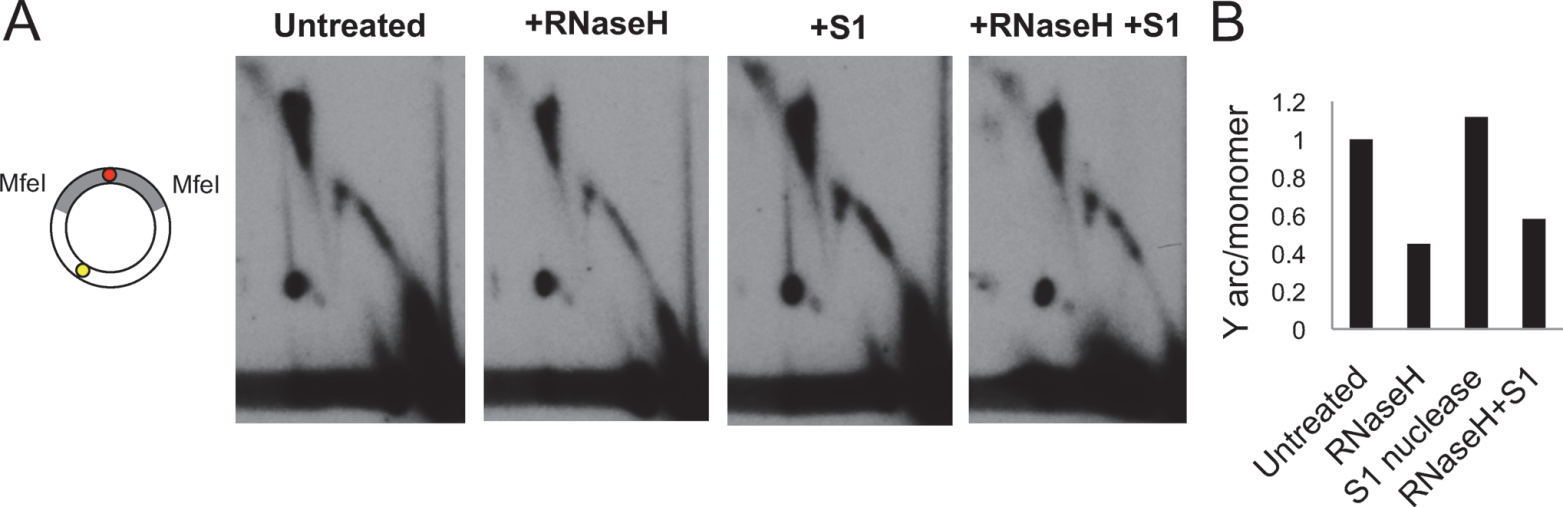
Advancing forks are engaged in strand-synchronous replication. MtDNA was treated with RNase H, S1 nuclease, or both, fractionated by 2DNAGE and blot-hybridized with a probe specific to the major-NCR, illustrated as in Fig. 1B. (A) untreated, MfeI-digested mtDNA (diagram at left); RNase H treatment diminishes replication intermediates, yet a full Y arc persists. Treatment by S1 nuclease alone does not alter migration of replication intermediates. A proportion of intermediates persist treatment with S1 nuclease after RNase H indicating a lack of RNA:DNA hybrid tracts in *C*. *elegans* mtDNA. (B) ImageJ-quantified mean hybridization intensity of the Y arc derived from the major NCR-containing region, relative to the monomer spot. See also S2 Fig. for similar analysis of the remainder of the genome.

We tested for strand-displacement and/or RITOLS intermediates by systematic treatment of mtDNA fragments, collectively representing the complete mitochondrial genome, with S1 nuclease, RNase H, or RNase H followed by S1 nuclease, with subsequent analysis by 2DNAGE. For each replicate, equal amounts of purified mitochondrial nucleic acid were electrophoresed for each treatment condition on the same gel, then transferred and hybridized in parallel with the same preparation of radio-labeled probe. For all mtDNA fragments analyzed, both the Y arc and X arc (cruciform spike) persisted after treatment with either S1 or RNase H alone (Fig. 2A, see also S2C–S2H Fig.). The intensity of the Y arc hybridization signal was modestly decreased by treatment with RNase H, yet the majority of fragment RIs remained following subsequent treatment with S1 nuclease (Fig. 2A; quantification in Fig. 2B) and, importantly, were not converted to any other structure, demonstrating distinct electrophoretic migration.

These experiments indicate that *C*. *elegans* RIs lack the extensive ssDNA character expected from strand-displacement synthesis. Furthermore, slow-moving Y arcs were not observed, and depletion of the Y arc signal after treatment with RNase H and S1 was no greater than with RNase H treatment alone (Fig. 2B). Thus RNase H failed to ‘unmask’ substantial ssDNA regions, as would be expected if extensive RNA:DNA hybrid tracts were present. These data are consistent with synchronous (or very near-synchronous) replication of the two mtDNA strands independent of a D-loop, and eliminate from further consideration both asymmetric and RITOLS-mode strand-displacement replication.

### *C*. *elegans* mtDNA replication intermediates are branched-circular lariats with linear concatemer synthesis products

The absence of theta-form, RITOLS and partially ssDNA strand-displacement intermediates led us to consider alternate DNA replication mechanisms. The detection of Y arcs, but not bubble arcs, by 2DNAGE of fragments derived from a circular template is consistent with a rolling circle replication (RCR) mechanism [19,20]. According to the RCR model, sustained elongation on a circular template produces linear DNA molecules greater than template unit-length that may become resolved to monomers in a variety of ways, or remain concatemeric linear networks [21]. A central prediction of the RCR model is the presence of “lariat” DNA forms *in vivo*. For *C*. *elegans* mtDNA, we hypothesized the occurrence of one-genome unit length circular templates, from which multimeric linear tails would extend. Alternatively, a second replication mode can be envisaged that would involve strand-invasion of a linear template by linear molecules, as occurs in some bacteriophages and the mtDNA of the fungus *Candida albicans* [22,23]. This alternative would predict Y-form RIs in the absence of bubble RIs, but not lariat structures.

**Fig 3:**
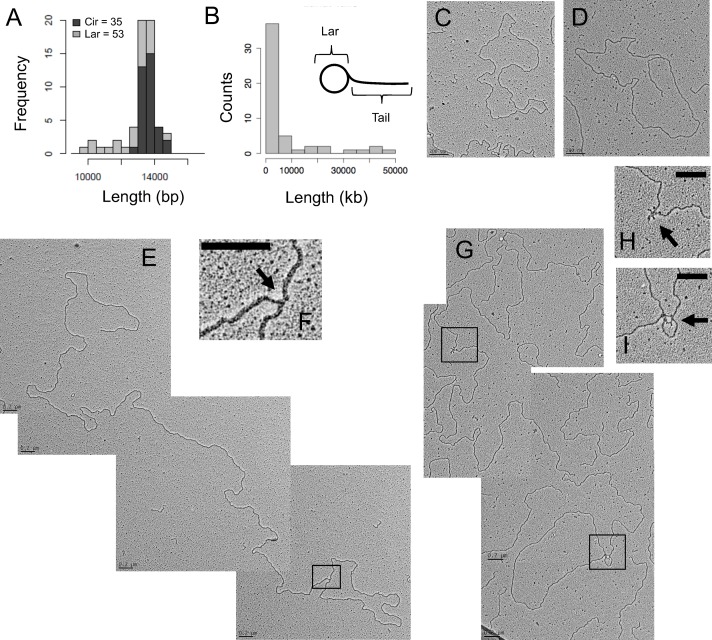
*C*. *elegans* mtDNA forms lariat-shaped rolling circle replication intermediates. RNase I-treated mitochondrial nucleic acid was spread on parlodion-coated copper grids and visualized by TEM. (A) Frequency distribution of estimated strand-lengths of monomeric circular mtDNAs (Cir) and the circular portion of lariat mtDNAs (Lar) in kb. (B) Frequency distribution of estimated tail lengths of lariat mtDNAs, in kb. (C) Representative electron micrograph of a double-stranded DNA monomer circle. (D) Representative electron micrograph of an mtDNA lariat. (E) Multimeric lariat molecule with one genome unit-length circle (1n; 13.8 kb) with concatemeric linear tail (3.5n; 48.2 kb). (F) Higher magnification of the apparently double-stranded circle-tail junction. (G) Lariat with partial ssDNA structure consisting of a genome unit-length circle (1n; 13.8 kb) and concatemer tail (2.6n; 35.7 kb). Magnification of the single-stranded regions along the tail (H), as well as at the tail-circle junction (I); arrows indicate ssDNA. Scale bars, 0.2 μm. Data generated from three independent mtDNA isolations.

To determine whether lariat molecules consistent with rolling circle intermediates were present, we directly examined *C*. *elegans* mtDNA using transmission electron microscopy (TEM). We observed both circular and branched-circular lariat molecules (Fig. 3, S1 Table). Most prominent were dsDNA circles with a mean measured length of 13.61 kb +/- .407 kb, consistent with the sequenced mitochondrial genome size of 13.794 kb [10] (Fig. 3A, B). Although *C*. *elegans* mtDNA has long been thought to be circular [10], based on its restriction map, to our knowledge this is the first evidence that non-replicating *C*. *elegans* mtDNA exists in a topologically circular form. As predicted by our 2DNAGE and bioinformatic analyses, none of these circular mtDNAs contained a visible displacement loop. Lariats with linear tails ranging from < 1 kb to 48.2 kb in length, i.e., more than three genome units, were the next most frequently observed class of molecules (Fig. 3C, D; S1 Table). The mean length of the lariat circular portion measured 13.64 kb (Fig. 3A). Fifty-six percent of lariat molecules appeared fully double-stranded at the circle-branch junction (Fig. 3E, F), though ssDNA tracts of less than ∼500 bases are not readily visible under the imaging conditions used.

While the X arc intermediates described above are intense on fragment 2DNAGE, their full structure, when undigested by restriction enzymes, is unknown. To further address the structure of the cruciform mtDNA, we isolated X-arc intermediates from a second-dimension gel on which ClaI/ApaI digested *C*. *elegans* mtDNA was fractionated. However, when spread for TEM, microfragments of agarose remained bound to the DNA, compromising the visualization of these molecules and precluding their further characterization. In non-fractionated spreads of purified mtDNA such forms would be indistinguishable from cases where two independent molecules are incidentally in contact.

Some linear molecules were observed by TEM. However, sub-genomic linear molecules were not detected by Southern blot of *C*. *elegans* mtDNA using any of the probes described in this study (S2 Table). We conclude that linear molecules on TEM grids are most likely contaminating nuclear DNA fragments, and therefore excluded them from the analysis summarized in S1 Table.

A subset of lariat molecules contained visible interspersed regions of collapsed secondary structure, considered diagnostic for ssDNA (Fig. 3G) [24]. Within individual molecules, these regions occurred at several different positions: the junction of the circular and linear tail portions of lariats, further along the linear branch only, or in both locations (Fig. 3H, I). Neither strand-displacement nor theta RIs were observed among the 1262 molecules analyzed by TEM, while lariats made up approximately 4% of mtDNA molecules, in line with previous reports of the proportion of replicating mtDNA in other species, e.g. mammalian cells and *Drosophila melanogaster* [3,25].

### The major non-coding region harbors cruciform mtDNA species

The high frequency of non-replicative circular monomers we detected by TEM suggested inter-conversion between the circular monomer and the lariat mtDNA forms. In the course of fragment 2DNAGE, we noted that the hybridization intensity of cruciform structures was most prevalent, relative to the monomer spot, in fragments harboring the 465 bp ‘major’ NCR (Fig. 1). This observation suggested that the formation of a site-specific cruciform structure could play a role in the maintenance of *C*. *elegans* mtDNA and/or the production of monomer circles [21,26].

**Fig 4:**
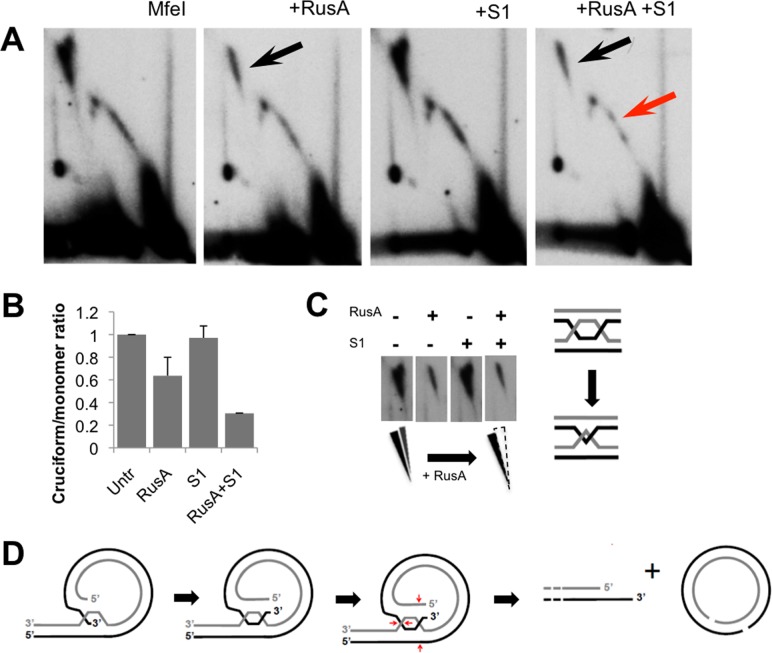
Prominent Holliday junctions and hemicatenanes indicate a recombination hotspot within the major non-coding region. 2DNAGE patterns of MfeI-digested mtDNA treated with RusA resolvase, S1 nuclease, or both; panels are representative of three independent experiments. (A) RusA treatment degrades the cruciform spike (black arrow), while S1 nuclease treatment alone does not effect migration of replication intermediates. RusA and S1 nuclease in combination further reduced the cruciform signal (black arrow) and deplete a subset of Y arc intermediates (red arrow). (B) ImageJ quantification of cruciform structure sensitivity to RusA, S1 nuclease, or both relative to the 1n monomer signal. Quantification represents the mean from three independent experiments +/- s.d.). (C) Left: Two partially overlapping spikes of X-shaped intermediates are visible in Fig. 4A; the rightmost cruciform spike displays enhanced sensitivity to RusA while the leftmost spike is insensitive, consistent with the presence of hemicatenanes. Right: Convergence of two cruciforms produces a hemicatenane structure. (D) Speculative model for lariat-to-monomer resolution involving strand invasion, crossover and resolution of four-way junctions.

We further addressed cruciform architecture by 2DNAGE following treatment with RusA, an *Escherichia coli* resolvase highly specific for Holliday junctions substrates in *in vitro* studies [27,28]. This analysis was performed using RusA alone or in combination with S1 nuclease. RusA treatment reduced the hybridization signal of the cruciform spike by 48% relative to the untreated controls, while S1 nuclease alone had no significant effect (Fig. 4A, B). Treatment with S1 after RusA further reduced the cruciform signal, and revealed a subclass of cruciforms resistant to both RusA and S1 that persisted after the combined treatment (Fig. 4A). On 2DNAGE these molecules formed a near-vertical spike (Fig. 4C), a migration pattern typical of hemicatenanes [29]. It has previously been reported that the collapse of adjacent four-way junctions produces resolvase-resistant hemicatenanes [29,30]. These findings are reminiscent of the RusA-resistant mtDNA cruciforms we observe; whether these X-junctional molecules are intermediates in the mechanisms of mtDNA RCR or monomer resolution awaits further investigation.of mtDNA RCR or monomer resolution awaits further investigation.

## Discussion

Taken together, our findings indicate that synthesis of *C*. *elegans* mtDNA proceeds by rolling circle replication. We propose that multiple replication cycles on single template circles generate lariat structures with multimer tails composed primarily, although not entirely, of dsDNA, which are subsequently resolved to monomer circles. Such conversion could potentially involve the formation and resolution of the cruciform species observed by fragment 2DNAGE (Fig. 4).

In the context of a rolling circle, each initiation event will give rise to multiple genome units, making the identification of a specific start site challenging. Neither the biochemical nor microscopic methods used here revealed a specific site of replication initiation, nor did our bioinformatic analysis identify molecular signatures thereof [12]. The data presented here do not exclude the possibility of replication initiation via site-specific nicking followed by strand invasion, or alternatively by homologous recombination. In such a case, the intensity of the cruciforms from ClaI-ApaI and MfeI-MfeI mtDNA fragment gels would be consistent with the NCR region containing a site-specific origin of such a type. It is worth noting, however, that the *C*. *elegans* NCR region contains an approximately 1 kb region of short repetitive elements with a potential to form complex secondary structures necessitating replication fork pausing and restart, or perhaps facilitate intermolecular strand exchange [10]. Our 2DNAGE analysis of mitochondrial nucleic acid isolated from the glp-4(bn2) mutant strain revealed a marked diminution or absence of both canonical replication intermediates and cruciform species (S2 Fig.). These data further imply that the cruciforms detected in wildtype animals are potentially involved in synthesis and/or resolution of mtDNA in the gonad.

The data presented herein do not directly address molecular recombination involving sequence-specific mtDNA strand exchange events in *C*. *elegans*. The occurrence of genetic recombination within or between mtDNA molecules in animal mitochondria is highly controversial [30,31], and there is currently no evidence for sequence-specific inter- or intra-molecular recombination of vertebrate mtDNA. Indeed, recent work has demonstrated that recombination is undetectable in the germline of mice segregating neutral mtDNA haplotypes, when tracked over 50 generations [32]. Sequence alterations suggestive of recombination have been detected in heteroplasmic mice carrying a deleterious allele [33], although the changes were detected at very low frequency and could plausibly have resulted from *in vivo* template switching during replication. Purified mtDNA both from mice and cultured mouse embryonic fibroblasts has been analyzed extensively by 2DNAGE, without the detection of prominent cruciform species such as those we infer for *C*. *elegans* [5,34]. In contrast, junctional mtDNA species have been detected by both 2DNAGE and TEM methods in human heart and brain[35,36].

Elsewhere within the metazoa, compelling evidence has accrued for recombination between maternal and paternal mtDNA haplotypes in the mussel *Mytilus* in which inheritance of the mitochondrial genome is doubly uniparental [37]. Moreover, PCR-based methods have also implied novel sequence organizations consistent with intramolecular recombination among short tandem repeat arrays in the mitochondrial genome of the plant parasitic nematode *Meloidogyne javanica* [38].

The fact that no animal mtDNA resolvases have been identified to date remains a major challenge to the mtDNA recombination concept [39]. We searched the *C*. *elegans* genome for plausible mitochondrially targeted homologues of known integrase or resolvase gene families using bioinformatic methods, without success. This raises the possibility that the resolution of rolling-circle replication intermediates to genomic monomers in *C*. *elegans* involves known proteins involved in worm mtDNA maintenance [9,36,37], which may have adopted novel molecular functions. While some mitochondrial proteins required for the maintenance of mtDNA copy number have been described in *C*. *elegans* [40–44], the functional architecture of the minimal mtDNA replisome remains to be elucidated. Future work describing the effect of manipulation of known factors on mtDNA replication intermediates by 2DNAGE could potentially reveal or exclude roles for conserved mtDNA maintenance factors in rolling circle replication.

Several of the known metazoan mtDNA maintenance factors are highly homologous to bacteriophage proteins, including the mtDNA polymerase POLG, RNA polymerase POLRMT, and TWINKLE helicase [3]. Moreover, antiviral drugs are commonly mitotoxic [3]. These observations raise the possibility that the genome of the ancestral endosymbiont may have been replicated by a phage-like RCR mechanism [3], of which the DNA replication system used in the *C*. *elegans* germline is a relic. The analogy with T7 replication is furthered by (i) the presence of sporadic ssDNA regions observed along lariat molecules (Fig. 3H), which are consistent with yet-to-be-completed and ligated gaps between lagging-strand synthesis products and (ii) looping at the lariat circle-to-tail junction (Fig. 3I) that could represent a single replisome engaged in coordinate synthesis of the leading and lagging strands, consistent with the 2DNAGE data presented here (Fig. 2A). If RCR was the ancestral mode of animal mtDNA replication, strand-displacement, RITOLS and other types of theta replication may represent taxon-specific derived mechanisms. They may represent different solutions to the challenge of maintaining genomic fidelity in the oxidative environment of the mitochondrion.

Rolling-circle replication of mtDNA has been described in the plant and fungal kingdoms [45–49]. Here we present the first report of RCR in a metazoan, furthering the ubiquity of this mechanism of mtDNA synthesis. Our findings differ from the descriptions of RCR in plants and fungi, in that the *C*. *elegans* mtDNA monomer circle remains the most common topology of non-replicative mtDNA. Among the fungi, replication of *Saccharomyces cerevisiae* mtDNA produces linear molecules, with circles present only transiently during replication; in contrast, mtDNA synthesis in *Candida albicans* is recombination driven, generating concatameric linear networks [23,50]. Intriguingly, the implied similarities between *C*. *elegans* and yeasts with respect to mtDNA replication mechanisms and the high proportion of junctional mtDNA intermediates mirror similarities in the patterns and rates of mtDNA mutation observed in these species [51]. These data raise the possibility these two phenomena are linked. Plant mitochondrial genomes are particularly complex, often consisting of a mix of branched, linear and circular topologies that may be many genome multimers in size, rendering monomer circles a rare occurrence [46]. *C*. *elegans* mtDNA topology is also distinct in one aspect from models of bacteriophage RCR, due to the apparent absence of linear mtDNA monomers as detected by Southern blot, which in phages may bear distinct (phage T7) or permuted (phage T4) ends [21,52].

It has been previously assumed that *C*. *elegans* mtDNA adheres to the strand-displacement replication mechanism in which the NCRs contain first- and second-strand origins [7,8]; here we demonstrate that this cannot be the case. Neither putative replication origin produces bubble-type intermediates that are clearly observed in multiple mammalian systems including cultured human cells [53]. Rather, junctional mtDNA species were the only detectable and identifiable structures observed specific to either of the NCRs. While the details of concatemer resolution remain to be determined, we suggest that the junctional intermediates identified here may represent termination/resolution structures, in which strand-invasion or branch migration arrest could occur in a site-specific manner, facilitated by the short repeats present in the major NCR [10]. Invasion by the unreplicated ssDNA 3’ end of the lariat tail at the major NCR sequence would create a triple-stranded structure not unlike initial events in DNA strand exchange. Subsequent migration of this junction would provide an opportunity for formation of a second four-way junction. Cleavage and resolution would then generate a gapped circular monomer and lariat tail with 3’ overhang, one genome unit-length shorter. Such a mechanism could also produce rare uni-circular multimers (see S1 Table) in which resolution does not occur at an adjacent concatemer.

This mode of resolution, while speculative, is consistent with two intriguing aspects of our results. First, a subset of Y arc RIs are sensitive to degradation by RusA (Fig. 4A), indicating that some Y-like forms in fact contain four-way junctions, as predicted by a strand-invasion model. Second, our 2DNAGE analysis of the 5 kb mtDNA region containing the major NCR demonstrated the presence of RusA-resistant cruciforms consistent with hemicatenanes, a DNA species which forms via the convergence of double Holliday junctions, or alternatively by replication fork stalling [54–56]. We did not observe molecules simultaneously involved in elongation and resolution by TEM, which would be consistent with this model. However, we note that if present *in vivo*, such molecules could possibly exceed 75 kb in size and therefore are likely to be fragile to DNA isolation techniques. The enzymes involved in putative site-specific resolution remain unknown. Other mechanisms can be envisaged which would exploit homologous recombination machinery documented to exist in mitochondria in at least some species[57–59], for example, involving site-specific DNA binding proteins and/or branch-migration driven by directionally acting helicases.

Whether RCR occurs elsewhere in the animal lineage remains to be explored. Unlike previous studies characterizing mtDNA RIs in organisms where both strands of mtDNA contain protein coding information, all protein-coding genes are transcribed from one *C*. *elegans* mtDNA strand (Fig. 1A), thus raising the possibility that RCR may be linked to the transcriptional architecture of the mitochondrial genome. Fortunately, the Nematoda are an excellent model system in which to test such hypotheses. The mtDNAs of many nematode species have been sequenced, revealing considerable architectural variation and enabling comparative studies in which the mtDNAs differ by gene amplification or inversion, scrambled gene orders, or translocation of the major NCR in the genome map [60]. As such, the phylum presents a powerful new model system for probing the relationship between mitochondrial genome architecture and replication mode.

*C*. *elegans* itself offers a compelling model for the study of rolling circle replication in animal cells from both mechanistic and genetic perspectives. MtDNA replication primarily occurs in the *C*. *elegans* germline, where high demand during gametogenesis likely requires efficient, yet prolific mtDNA synthesis [1]. Since RCR is the only replication mode that can be detected, it must be sufficient to meet this demand despite the small percentage of molecules replicating at any particular time; we note that mtDNA multimers resulting from RCR can potentially resolve to several copies of the mitochondrial genome (Fig. 3), in contrast to theta replication, which produces only two daughter mtDNA molecules. We anticipate that the future characterization of factors involved in this process will provide many new insights into animal mtDNA maintenance, the evolution of replication mechanisms, and possibly even the pathological derangement of mtDNA synthesis in humans.

## Materials and Methods

### Nematode maintenance and mtDNA isolation

*Caenorhabditis elegans* strains N2 Bristol and mutant *glp-4(bn2)I* were obtained from the *Caenorhabditis* Genetics Center (CGC; Minneapolis, USA) and maintained as described [15,61]. For wildtype N2 animals grown in liquid culture, a culture sample was removed every 24 hours for monitoring and the turbidity of the culture tested to ensure ample E. coli OP50 were present. Culture samples were visually also checked for developmentally arrested or dauered larvae that could indicate bacterial depletion—none were observed. N2 and *glp-4* (bn2)I animals grown on plates were fed on a lawn of E. coli OP50 as described [62]. Nematodes were actively growing and feeding on E. coli OP50 until immediately prior to mitochondrial isolation. For preparation of intact mitochondria, nematodes were collected, dounce-homogenized and subjected to differential centrifugation to generate a crude mitochondria-enriched fraction; further enrichment was achieved by sucrose-step gradient centrifugation (1:1.3 M sucrose), with all procedures conducted at 4°C [25]. Lysis and DNA extraction protocols for *C*. *elegans* were adapted from studies in which fragile mtDNA replication intermediates have previously been successfully isolated intact, for analysis by both 2DNAGE and TEM [18,63,64]. Briefly, freshly isolated mitochondria were lysed with 1% SDS in the presence of 200 mg Proteinase K and extracted by phenol-chloroform and ethanol precipitation; mtDNA was washed in ethanol and re-suspended in Tris-EDTA pH 7.6 for further manipulation.

### Enzymatic treatments

Treatments (restriction endonucleases, RNases, other nucleases) were executed according to manufacturer instructions; RusA treatment was as described [28].

### 2D neutral agarose gel electrophoresis and blot hybridization

2DNAGE, blot-transfer and probe hybridization were carried out as previously described [25,65]; see S2 Table for genomic locations and sequences of mtDNA probes. For all 2DNAGE panels, four-hour exposures are presented. For detailed information on probes see Supplemental Experimental Procedures.

### Transmission electron microscopy

Aliquots of RNase I-treated mitochondrial nucleic acid were mounted directly on parlodion-coated copper grids following the Kleinschmidt method and imaged as described [24,66]. Molecule lengths were measured in Gatan DigitalMicrograph and calibrated by measurement of a co-spread 3.5 kb pglGAP plasmid. Each mtDNA molecule was measured 3 times to obtain mean values as reported in Fig. 3.

### Bioinformatic analysis

Cumulative GC skew analysis was carried out as described [23] using a custom *R* script.

## Supporting Information

S1 Fig 2DNAGE analysis of replication intermediates spanning non-coding regions of the *C*. *elegans* mitochondrial genome. Methods are as in Fig. 1. Arcs indicative of replication initiation bubbles were not detected on overnight, two day (shown here), four day or eight day autoradiographic exposures. Replication intermediates identified in the 465 bp ‘major’ NCR excised by (A) MfeI or (B) ClaI/ApaI digests; (C) the 104 bp ‘minor’ NCR excised by BsrGI/ClaI digests; or the coding region only (D) ApaI/BsrGI) digests. (E) Anticipated position (dotted red line) of bubble intermediates after resolution by 2DNAGE. (F) Restriction map of *C*. *elegans* mtDNA depicting sites used to linearize mtDNA after cleavage with (G) NdeI, (H) BsrGI, or (I) MbiI. Linearized replication intermediates were subsequently resolved by 2DNAGE. After blot-hybridization using a ^32^P-labeled nd5 probe, replication intermediates were identified by autoradiographic detection. Arcs of predicted replication intermediates are diagrammed in (J); clockwise from top right: uncut circles; Y arc of advancing replication forks; bubble arc; 1 genome unit length linear arc; unknown spot; and cruciforms. One explanation for the migration of the unknown spot, which begins at the apex of the Y arc and proceeds towards the cruciforms, would be an irregular Y form (upper right) wherein one arm of the Y is greater than 0.5n (>50% unit length), in contrast to a classic Y-form (lower right) in which the apex of the arc corresponds to precisely 0.5n. We suggest irregular Y forms could indicate the presence of replication intermediates > 2n. This irregular Y spot is specific to 2DNAGE of the linearized genome and does not appear when mtDNA sub-genomic fragments are analyzed by 2DNAGE. Panels shown are representative of two independent experiments. (K) The cumulative GC skew profile of *C*. *elegans* mtDNA was calculated over 200 bp windows at 50 bp sliding intervals using custom R scripts. The genomic locations of the minor NCR (yellow arrow) and major NCR (red arrow) are marked by gray dotted lines. Neither local minima, indicative of site-specific replication initiation, nor local maxima indicative of site-specific termination were present.
(PDF)

S2 Fig Lack of theta-type mtDNA replication initiation intermediates from *glp-4(bn2)* or wildtype N2 nematodes. Comparison of replication and recombination intermediates from the 465 bp ‘major’ NCR excised by MfeI in *glp-4* mutants grown at 16°C (A) and 25°C (B), after 1 hour (left panel) or 96 hour (right panel) exposure of hybridized mtDNA to film, demonstrating lack of ongoing mtDNA replication in gonadless worms. Cruciform spike RIs are readily visible in sample from animals cultured at permissive temperature, i.e. 16°C, and less so in animals cultured at 25°C. RIs specific to the minor NCR region (C-E) or the coding region only (F-H) were identified by autoradiographic detection with the probes indicated before (C, F) or following treatment with RNase H (D, G) or S1 nuclease (E, H). For all digests, a full Y arc persisted after both RNAse H and S1 nuclease treatments. Panels shown are representative of three independent experiments.
(PDF)

S1 Table Summary of 1262 non-linear mtDNA molecule topologies observed by transmission electron microscopy. Molecules are grouped into monomer (top 2 rows) and multimer (subsequent rows) forms.
(PDF)

S2 Table Genomic locations and nucleotide sequences of *C*. *elegans* mtDNA probes for Southern hybridization. (DOCX)
