# Supplementary figures and images for: A Rolling Circle Replication Mechanism Produces Multimeric Lariats of Mitochondrial DNA in Caenorhabditis elegans
Source: PLoS Genet. 2015 Feb 18;11(2):e1004985. doi: 10.1371/journal.pgen.1004985 (PMC4334201; doi:10.1371/journal.pgen.1004985)

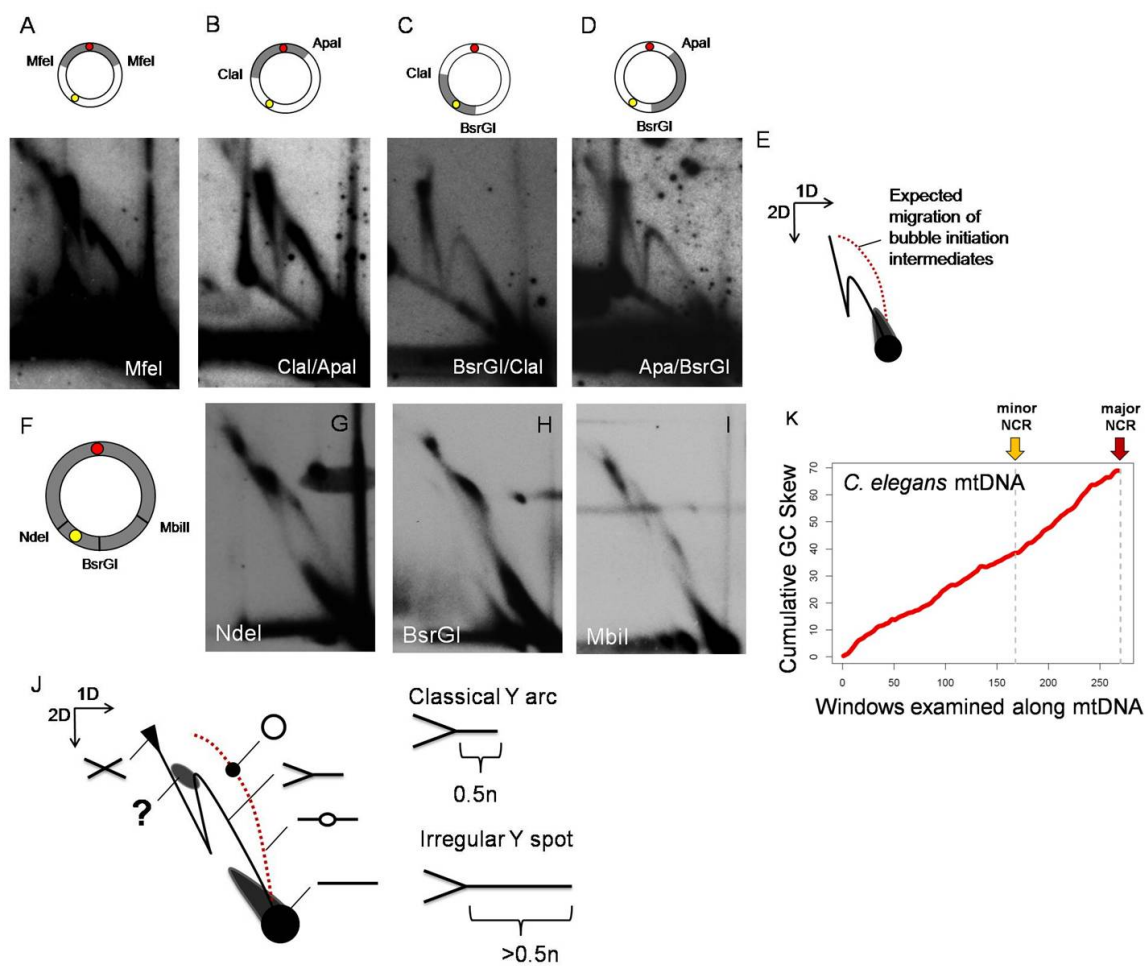

**Figure S1.**

Supplement: S1 Fig — Methods are as in Fig. 1. Arcs indicative of replication initiation bubbles were not detected on overnight, two day (shown here), four day or eight day autoradiographic exposures. Replication intermediates identified in the 465 bp ‘major’ NCR excised by (A) MfeI or (B) ClaI/ApaI digests; (C) the 104 bp ‘minor’ NCR excised by BsrGI/ClaI digests; or the coding region only (D) ApaI/BsrGI) digests. (E) Anticipated position (dotted red line) of bubble intermediates after resolution by 2DNAGE. (F) Restriction map of C. elegans mtDNA depicting sites used to linearize mtDNA after cleavage with (G) NdeI, (H) BsrGI, or (I) MbiI. Linearized replication intermediates were subsequently resolved by 2DNAGE. After blot-hybridization using a 32P-labeled nd5 probe, replication intermediates were identified by autoradiographic detection. Arcs of predicted replication intermediates are diagrammed in (J); clockwise from top right: uncut circles; Y arc of advancing replication forks; bubble arc; 1 genome unit length linear arc; unknown spot; and cruciforms. One explanation for the migration of the unknown spot, which begins at the apex of the Y arc and proceeds towards the cruciforms, would be an irregular Y form (upper right) wherein one arm of the Y is greater than 0.5n (>50% unit length), in contrast to a classic Y-form (lower right) in which the apex of the arc corresponds to precisely 0.5n. We suggest irregular Y forms could indicate the presence of replication intermediates > 2n. This irregular Y spot is specific to 2DNAGE of the linearized genome and does not appear when mtDNA sub-genomic fragments are analyzed by 2DNAGE. Panels shown are representative of two independent experiments. (K) The cumulative GC skew profile of C. elegans mtDNA was calculated over 200 bp windows at 50 bp sliding intervals using custom R scripts. The genomic locations of the minor NCR (yellow arrow) and major NCR (red arrow) are marked by gray dotted lines. Neither local minima, indicative of site-specif [file pgen.1004985.s001.pdf]

**Figure S2.**

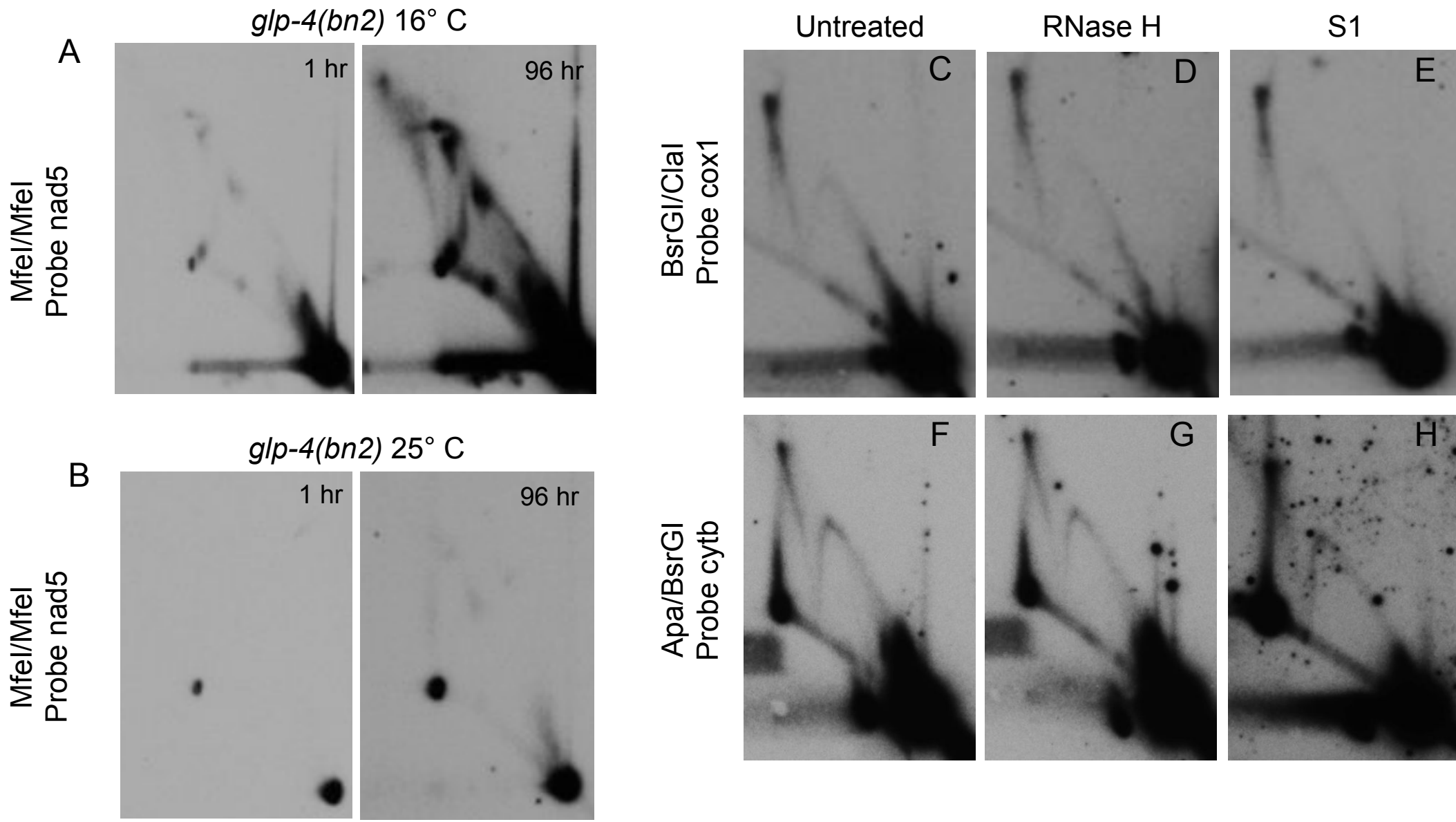

Supplement: S2 Fig — Comparison of replication and recombination intermediates from the 465 bp ‘major’ NCR excised by MfeI in glp-4 mutants grown at 16°C (A) and 25°C (B), after 1 hour (left panel) or 96 hour (right panel) exposure of hybridized mtDNA to film, demonstrating lack of ongoing mtDNA replication in gonadless worms. Cruciform spike RIs are readily visible in sample from animals cultured at permissive temperature, i.e. 16°C, and less so in animals cultured at 25°C. RIs specific to the minor NCR region (C-E) or the coding region only (F-H) were identified by autoradiographic detection with the probes indicated before (C, F) or following treatment with RNase H (D, G) or S1 nuclease (E, H). For all digests, a full Y arc persisted after both RNAse H and S1 nuclease treatments. Panels shown are representative of three independent experiments. (PDF) [file pgen.1004985.s002.pdf]
