# Supplementary material for: A Rolling Circle Replication Mechanism Produces Multimeric Lariats of Mitochondrial DNA in Caenorhabditis elegans
Source: PLoS Genet. 2015 Feb 18;11(2):e1004985. doi: 10.1371/journal.pgen.1004985 (PMC4334201; doi:10.1371/journal.pgen.1004985)
Supplement: S1 Table — Molecules are grouped into monomer (top 2 rows) and multimer (subsequent rows) forms. (PDF) [file pgen.1004985.s003.pdf]

Supplementary Table 1.

Summary of 1262 mtDNA molecule topologies observed by transmission electron microscopy. Molecules are grouped into monomer (top 2 rows) and multimer (subsequent rows) forms.

**Molecular species**		**N of 1262**	**%**
Relaxed double-stranded DNA: one genome unit length circles	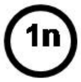	1104	87.5
Circles with complex topology: supercoiled, gapped or twisted	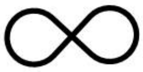	91	7.2
Branched-circular lariats:<br>Double-stranded DNA (35)<br>Partially single-stranded DNA (18)	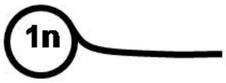	53	4.2
Branched circular: non-lariat	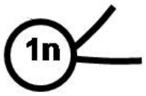	4	0.3
Multimer circles: unicircular or potential recombination intermediates	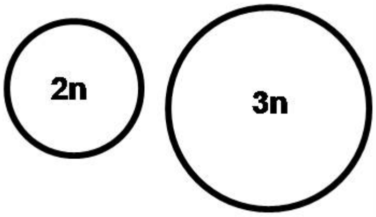	10	0.7
