# Supplementary material for: A Rolling Circle Replication Mechanism Produces Multimeric Lariats of Mitochondrial DNA in Caenorhabditis elegans
Source: PLoS Genet. 2015 Feb 18;11(2):e1004985. doi: 10.1371/journal.pgen.1004985 (PMC4334201; doi:10.1371/journal.pgen.1004985)
Supplement: S2 Table — (DOCX) [file pgen.1004985.s004.docx]

**Supplementary Table S2.**

Genomic locations and nucleotide sequences of *C. elegans* mtDNA probes for Southern hybridization.

**
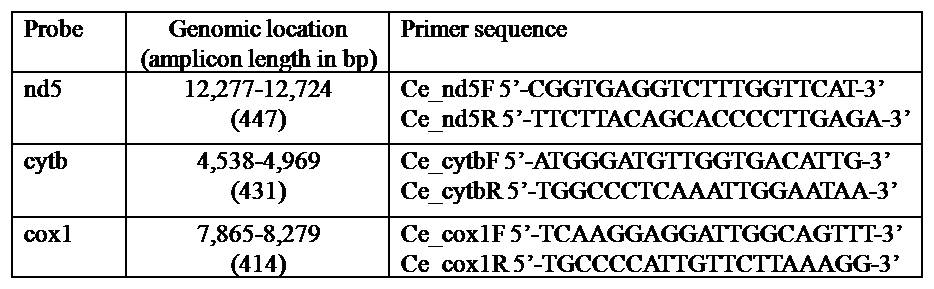
**
